# Establishment of a population pharmacokinetics model of vancomycin in 94 infants with septicemia and its application in individualized therapy

**DOI:** 10.1186/s40360-021-00489-8

**Published:** 2021-05-04

**Authors:** Zhiling Li, Hongjing Li, Chenyu Wang, Zheng Jiao, Feng Xu, Huajun Sun

**Affiliations:** 1 Department of Pharmacy, Shanghai Children’s Hospital, Shanghai Jiao Tong University, No. 355 Luding Road, Putuo District, Shanghai, 200062 China; 2 Department of Pharmacy, Shanghai Chest Hospital, Shanghai Jiao Tong University, Shanghai, China; 3 Fengxian Hospital, Southern Medical University, Shanghai, China

**Keywords:** Infants septicemia, Vancomycin, Population pharmacokinetic, Monte Carlo, Individualized administration

## Abstract

**Background:**

We aim to develop a population pharmacokinetics (PopPK) model of vancomycin for the treatment of septicemia in infants younger than one year. Factors influence of the PK was investigated to optimize vancomycin dosing regimen.

**Methods:**

The nonlinear mixed effects modelling software (NONMEM) was used to develop the PopPK model of vancomycin. The stability and predictive ability of the final model were assessed by using normalized prediction distribution errors (NPDE) and bootstrap methods. The final model was subjected to Monte Carlo simulation in order to determine the optimal dose.

**Results:**

A total of 205 trough and peak concentrations in 94 infants (0–1 year of age) with septicemia were analyzed. The interindividual variability of the PK parameter was described by the exponential model. Residual error was better described by the proportional model than the mixed proportional and addition models. Serum creatinine concentration and body weight are the major factors that affect the PK parameters of vancomycin. The clearance was shown to be higher when ceftriaxone was co-treated. More than two model evaluation methods showed better stability than the base model, with superior predictive performance, which can develop individualized dosing regimens for clinical reference. Through prediction of final model, the trough concentration was more likely < 5 mg/L when a routine dose of 10 mg/kg is administered every 6 h to 3–9-month-old infants. Therefore, the dose should be increased in the treatment of infant septicemia.

**Conclusions:**

The stable and effective PopPK model of vancomycin in Chinese infants with septicemia was established. This model has satisfactory predictive ability for clinically individualized dosing regimens in this vulnerable population.

## Background

Over the last decades, the most frequent causative agents of septicemia in neonates have been reported to be the Gram-positive cocci infections, especially methicillin-resistant *Staphylococcus aureus* (MRSA) [1]. Vancomycin is widely used in clinical scenarios as the antimicrobial therapy for targeted or empiric treatment of neonatal sepsis [2]. It is the first glycopeptide antibiotic with triple mechanisms, inclusive of inhibiting the synthesis of cell walls and RNA in the cytoplasm, and altering membrane permeability in bacteria [3]. However, there remain numerous disputes regarding the current individualization of vancomycin dose in clinical practice. Even with the routine dose of vancomycin in newborns admitted in the intensive care unit, we are still unable to easily obtain trough and peak plasma concentrations of 5 to 10 mg/liter and 20 to 50 mg/liter in neonate patients [4].

Vancomycin-associated nephrotoxicity is a critical appraisal of risk with high-dose therapy for infants and adults [5]. Nevertheless, the immaturity of physiological processes and organ functions, such as liver’s and kidney’s, predisposes infants to disparate drug disposition and responses compared with that in adults [5, 6]. It is especially important to avoid exposure to unnecessarily high peak or trough concentrations of vancomycin in the premature newborn. The pharmacokinetic (PK) and pharmacodynamic (PD) parameters constantly change across the pediatric groups. The disposition and response of infants are not only different from that of other aged children but also widely vary within themselves [6, 7]. These issues along with the lack of pharmacokinetics data on infants lead to remarkable difficulty in establishing the regimen for infants. Based on the assessment of population PK models, population PK parameter estimates are less biased than those obtained using the naive and standard two-stage approaches. Thus, the NONMEM software is the most widely used software for the characterization of population PK in clinical practice in order to solve the problem [8] . To date, vancomycin has been one of the most studied antibiotics using the PopPK in neonates and adults [9]. Approaches to vancomycin clinical dosing were developed on basis of the PK parameters of individuals [10]. The method does not only conduct statistical analysis using the sparse uneven data for each patient with a few samplings, but also integrate gestational age, gestational weeks, weight, and other important data of infants for PK data processing, which suitable for infants [10, 11]. Our previous investigation characterized the PK parameters of neonates [12], but the optimal use of vancomycin in infants need to be conducted continually. It is challenging to develop and validate a model that prospectively predicts individual exposure in infants and further optimize dosing regimens. Vancomycin is always used to combine with other antibiotics such as ceftriaxone and meropenem. In order to know whether drug interactions influence the blood concentration of vancomycin, this study aims to identify individual factors that affect drug interactions of ceftriaxone that subsequently influences variability and to establish dosing regimens for infants in China.

In this study, the PK parameters that affect the PK of vancomycin were completely elucidated, and the PopPK of vancomycin in infants (0–1 year of age) with septicemia in a Chinese population was developed. Based on the PopPK model, we screened for factors that might significantly influence the PK of vancomycin and provide a reference for the individualization of treatment with vancomycin in clinical practice. Our work would potentially reduce the incidence of adverse reactions originated from the use of vancomycin and improve efficacy and safety.

## Methods

### Patients and data collection

A total of 94 Chinese infants who received vancomycin for the treatment of septicemia at a grade A special hospital between January 2009 and December 2015 were enrolled. The following data were collected: blood concentration of vancomycin, daily vancomycin dose, demographic characteristics of patients, blood examination results, hepatic and renal function data, and co-administered medicines. Serum vancomycin concentration was determined by chemiluminescence analysis. Demographic and clinical data of infants are summarized and presented in Table 1.

**Table 1:** Demographic and Clinical Data of Infants

Characteristics	Mean ± SD	Median (range)
The number of infants (M/F)	94 (58/36)	
Samples	205	
Age (d)	67.14 ± 80.85	88.5 (1–345)
Weight (kg)	4.686 ± 2.57	4 (1.4–18)
Height (cm)	54.29 ± 8.28	52 (37–78)
Gestational age (week)	37.18 ± 3.71	39 (25.7–41.4)
Correct gestational age (week)	46.44	43 (31.14–83.07)
Birth weight (g)	2978 ± 815.49	3200 (850–4400)
Creatinine levels (μmol/L)	19.91 ± 7.45	18.25 (5.5–50)
Creatinine clearance (ml/min/1.73m^2^)	111.1 ± 49.71	120 (21.46–280)
Daily dose (mg/day)	74.69 ± 44.95	60 (20–200)
Observed concentration (ug/ml)	13.35 ± 10.82	10.6 (3.31–51.93)
ALT (U/L)	29.63 ± 27.14	20 (3–156)
AST (U/L)	48.2 ± 35.82	34.5 (13–241)
BUN (mmol/L)	3.11 ± 1.83	2.75 (0.6–9.5)
The total protein (g/L)	52.59 ± 7.95	53 (32.4–78)
Albumin (g/L)	34.23 ± 5.37	35 (18.45–43)

Using the fluorescence polarization immunoassay method with the AxSYM system, serum vancomycin concentrations were measured as part of the therapeutic drug monitoring activity in the hospital. The assay sensitivity limit was 2.00 mg/L. Coefficients of variation were 4.26, 2.94, and 4.06% at concentrations of 7.0, 35.0, and 75.0 mg/L, respectively (package insert, Axsym system, Abbot Laboratories, AbbotPark, IL, USA).

### PopPK modeling

The PopPK model was developed using the NONMEM version 7.2 (Icon Inc., PA, the USA), Fortran compiler: Intel Fortran XE 2011 Update 13 (Intel Corp, CA, the USA),Wings for NONMEM (version 7.2, Nick Holford, University of Auckland, Auckland, New Zealand, http://wfn.sourceforge.net), Xpose (version 4.2.1, Department of Pharmaceutical Biosciences at Uppsala University), R package (version 2.15.1, http://www.r-project.org), Perl-speaks-NONMEM version 3.5.3 (http://psn.sf.net), Perl (ActivePerl-5.14.2, 64 bit), and Pirana (version 2.7.0, http://www.pirana-software.com). NONMEM was used to employed pharmacokinetic analysis. Based on individual dataset, population values of the PK parameters were considered as fixed-effect parameters. Inter- and intra-individual variabilities were estimated as random effects.

205 trough and peak about vancomycin concentrations versus time were fitted to both one- and two-compartmental models with first-order elimination. The most suitable compartmental model was determined to be specified to NONMEM by ADVAN1-TRANS2 or ADVAN3-TRANS4 subroutines. By using these model specifications, the fixed-effect PK parameters were directly estimated. Total body clearance (CL) and volume of distribution (V) are for the one-compartmental model, while CL, volume of distribution of the central compartment (V_1_), intercompartment clearance (Q), and volume of distribution of the peripheral compartment (V_2_) for the two-compartmental model. Additive, proportional, and exponential error models were tested to describe inter- and intraindividual variabilities.

In preliminary screening phase, each of covariates that significantly improved the predictive ability of the basic model would be included. Observed concentration–time profile was used to compare. The influence of covariates on the PK parameters of vancomycin as follows: weight, age, sex, serum creatinine (SCR) concentration, blood urea nitrogen, creatinine clearance rate [CLCR, using the Modification of Diet in Renal Disease/MDRD4 equation [10] and Cockcroft–Gault (C–G) equation [13]], serum albumin concentration, aspartate transaminase and alanine transaminase levels, and concomitant drugs (ceftriaxone, meropenem, gentamicin, furosemide, ibuprofen, and dexamethasone). The significant covariates were then cumulatively added (forward stepwise fashion) to the model in the order of their contribution in reducing the objective function value (OFV, − 2 log likelihood difference) in the preliminary analysis until no further reduction in OFV is observed. Finally, back elimination was conducted to eliminate any unnecessary covariate from the full regression model in the descending order of their contribution to the change in OFV.

The best compartmental model (one vs. two compartments), error model (additive vs. proportional vs. combined error models), and the retention of covariate (s) in the model were determined with the statistical significance of the model and were evaluated via the likelihood ratio test using the minimum value of the OFV, as produced by the NONMEM program. Changes in OFV of > 6.63 and > 9.21 were considered significant based on the χ^2^ distribution with degree of freedom (df) = 1 and 2 (both *P* < 0.01), respectively [14].

Other diagnostic criteria were a reduction in the unexplained interindividual variability for the associated PK parameters and an improvement in the graphic diagnostic model. Graphics were obtained using plotted observed vs. predictive concentrations and observed vs. weighted residuals (predicted minus observed concentrations and weighted by standard deviation). First-order conditional estimation interaction was utilized to estimate the PopPK parameters. Covariates were screened according to the stepwise method, and the final PopPK model of vancomycin was then established by backward elimination. The exponential model was used to describe interindividual variability of the PK parameters. Residual error was better described by the exponential model than the mixed exponential and addition models.

### Covariate screening

Before covariate analyses, any correlation between the covariates (correlation coefficient > 0.5) should be examined, and the study should only choose one for the analysis. Using the Xpose 4 program package of the *R* language to draw each co-distribution map, it was examined whether the distribution of data is normal [15]. Parameter values of the final model is shown in Table 2 and screening process was summarized in Table 3.

**Table 2:** Parameter Value of the Final Model

Parameter	Definition	Estimates	RSE (%)	95% confidence interval
CL	clearance	10.3	29.60%	4.322–16.278
V	distribution volume	50.6	7.50%	43.211–57.989
Θ1	weight coefficient on CL	1.06	9.40%	0.865–1.255
Θ2	Serum creatinine coefficient on CL	−0.315	20.70%	−0.443--0.187
Θ3	Co-therapy with ceftriaxone coefficient on CL	1.46	16.70%	0.982–1.938
η1	Between-subject variability of Clearance	0.145	27.70%	
ε1	Proportional within-subject variability	0.194	16.10%

**Table 3:** Model Selection Process^a^

Model	Description & main characteristics	OFV value	△OFV value	Whether or not included
1	One compartment model	1098.753	0	YES
2	One compartment model, ETA was not estimated on V	1102.375	3.622	NO
Forward inclusion process				
3	Add WT on CL	1000.624	−69.045	YES
4	Add SCR on CL	988.23	−12.394	YES
5	Add co-therapy with ceftriaxone on CL	978.401	−9.829	YES
Backward elimination process				
6	Remove WT on CL	1016.409	44.627	YES
7	Remove SCR on CL	987.288	14.602	YES
8	Remove co-therapy with ceftriaxone on CL	981.069	8.187	YES

^a^This is a standard stepwise procedure for screening covariates in popPK analysis

### Model evaluation

Internal evaluation is the method in which original modeling data are used in resampling technology and in the establishment of a validation dataset to validate the model. The normalized prediction distribution errors (NPDE), and bootstrap methods were used to test the model [16]. The results of boostrap in final model were summarized in Table 4. The stability and predictive ability of the final model were evaluated using NPDE, bootstrap method, and external data. The results of the model average prediction error (MPE) and average absolute error (MAE) calculated by the external evaluation method are shown in Table 5.

**Table 4:** Bootstrap Results of Final Model

Percentiles	OFV	CL	V	WT	SCR	DC	BSV_CL	ERR1
*medians (50%)*	964.99	11.01	50.38	1.06	−0.34	1.40	0.14	0.19
*0.50%*	811.46	5.17	40.37	0.82	−0.66	0.89	0.03	0.11
*2.50%*	844.48	5.93	43.07	0.87	−0.54	0.97	0.05	0.13
*5%*	863.64	6.51	44.41	0.90	−0.49	1.01	0.06	0.14
*95%*	1079.97	17.96	56.70	1.25	−0.22	1.75	0.21	0.25
*97.50%*	1100.29	22.17	57.74	1.31	−0.20	1.79	0.23	0.27
*99.50%*	1149.60	28.42	60.35	1.40	−0.08	1.91	0.26	0.31

**Table 5:** Prediction Ability of the Basic and Final Models

Evaluation	Final model	Basic model
MPE (%)	21.306	43.116
SPE (%)	63.627	101.701
MAE (%)	54.825	74.428
RMSE (%)	65.647	108.211
MDPE (%)	14.451	33.177
MDAE (%)	46.361	48.575
MBA (%)	4.498	10.195
SDBA (%)	55.400	70.967

### Monte Carlo simulation

The final model was subjected to Monte Carlo simulation to determine the optimal dose under which the drug concentration of a certain patient can be controlled within the reference concentration. According to the range of the commonly used dose in infants and factors that set the scene, data files of each scene were written. Based on the PopPK model of vancomycin, data files of each scenario were simulated using the Monte Carlo method, and data of 5000 were generated, which were completed by the ONLYSIMULATION and SUBPROBLEMS modules in the NONMEM software SIMULATION.

## Results

### Basic model

This study established the PopPK model according to results of the conventional therapeutic drug monitoring (TDM), which only had the fixed sampling point trough and peak concentrations. Data on vancomycin concentrations versus time were fitted to both one- and two-compartmental models, respectively. Because sparse sampling method, there is not available data enough to establish a two-compartmental (Table 3). The results preferred the one-compartmental model as more suitable model to describe this set of data.

The additive, proportional, and exponential error models were tested to describe inter- and intra-individual variabilities. Interindividual variability was estimated using an exponential error model. Because of the extremely large relative standard deviation  in the interindividual variation  of V, which was not allowed, the ETA test was not conducted. Bootstrap is used to estimate statistical variance and interval estimation of the statistical method, which could be used to estimate the confidence interval of parameter values of 2.5–97.5%. If parameter values of the 95% confidence interval include 0, then the parameter estimates are not reliable.

For the residual variance model, the mixed model of proportional and additive errors was examined, and the proportional residual model is adopted. 

### Covariate Model*s*

**Fig. 1:**
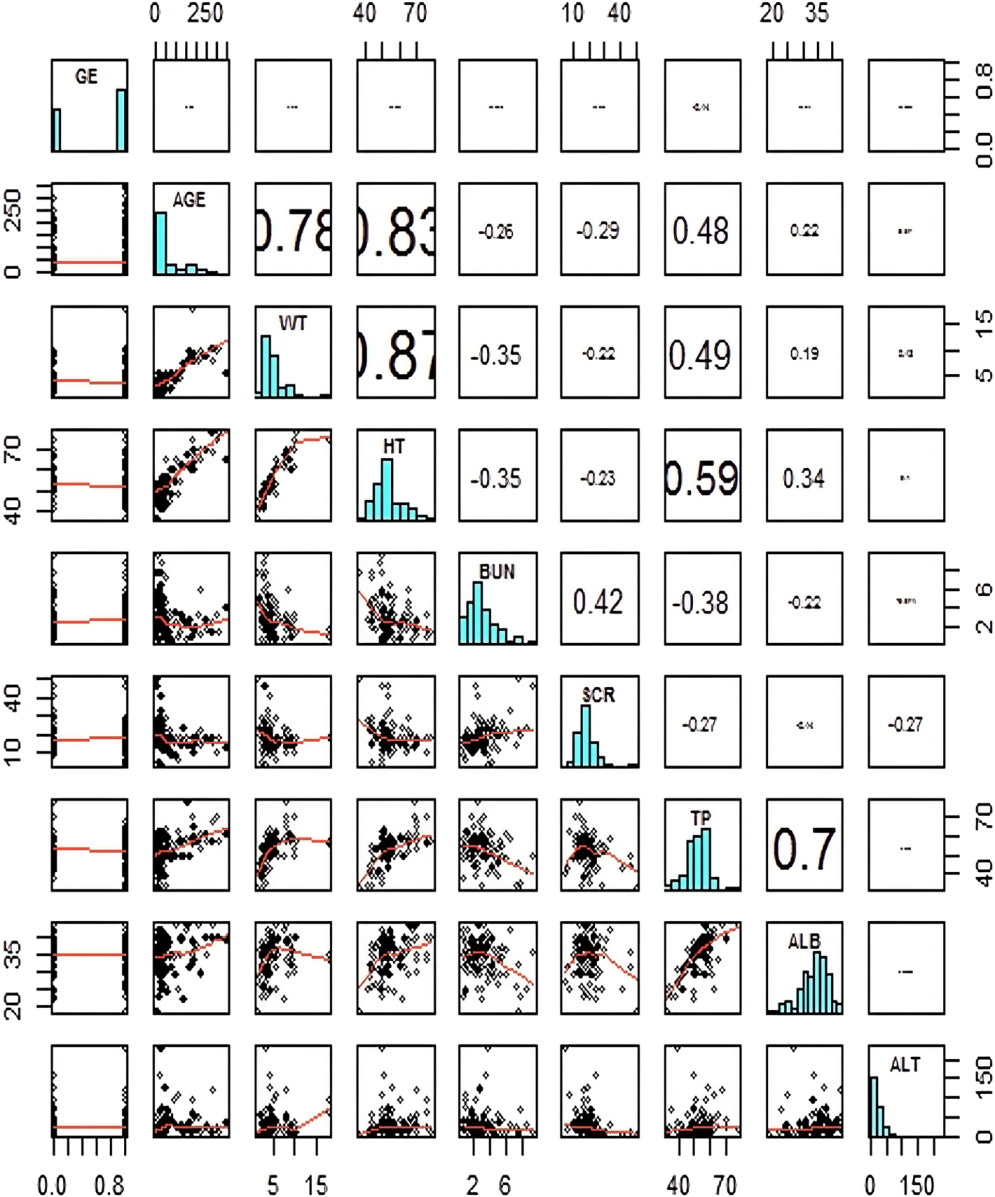
Correlation Analysis of Covariates in Infants

As depicted in Fig. 1, data obtained in this study had significant differences between the newborns and infants. The distribution of some covariates was not normal, including body weight, age, and SCR. Because the relationship between the clearance rate and body weight was obviously different at different phages, the method of subsection and different velocities should be adopted. Because the OFV value after adding weight generally decreased by > 100, body weight was considered an important covariate that influences the clearance rate and should be retained. Results of the second method showed that the correction of the prediction error was > 50%, which did not meet the requirements, and results of the first method had good stability. Thus, model 2 should be chosen for the next step of modeling.

First, based on the first method model, SCR concentration and CLCR were added to the CL as an impact factor; then, the two covariates were compared to identify the one that could better describe the CL. Results showed that OFV values decrease after the addition of SCR concentration and CLCR, and the OFV of the multiplication method would decrease more than the addition method. Because the OFV of the two models were similar and creatinine value was more convenient to obtain, which is much useful in future clinical development, the model for SCR as the covariate should be chosen.

The effects of concomitant drugs on clearance were investigated. The OFV of meropenem and ceftriaxone covariates decreased by > 3.84. Thus, meropenem and ceftriaxone should both be incorporated. The OFV of meropenem elimination decreased < 6.635 (*P* > 0.01), which was not statistically significant. Meanwhile, combination with meropenem neither had an effect on CL (< 20%) nor had clinical significance. Thus, meropenem should be removed and other covariates should be retained.

### Final model

The final regression model was as follows:

$$ CL\left(\frac{L}{hr}\right)=10.3\times {\left(\frac{\mathrm{WT}\left(\mathrm{kg}\right)}{70}\right)}^{1.06}\times {\left(\frac{\mathrm{SCR}\left(\mu \mathrm{mol}/\mathrm{L}\right)}{20}\right)}^{-0.315}\times {1.46}^{DC} $$

$$ V(L)=50.6\times \left(\frac{\mathrm{WT}\left(\mathrm{kg}\right)}{70}\right) $$

DC = 1 when incorporated with ceftriaxone, then DC = 0.

SCR and body weight were the major factors influencing the PK parameters of vancomycin. The clearance would be larger when ceftriaxone is incorporated.

Compared with the basic model, the external validation of the final model showed that the fitting degree of the predicted and measured values obviously improved, and the individual value average prediction errors of the final model was smaller but higher in the forecast precision. Then, the final model showed better fitting effect after adding the fixed-effect factors (Table 2).

### Model evaluation

**Fig. 2:**
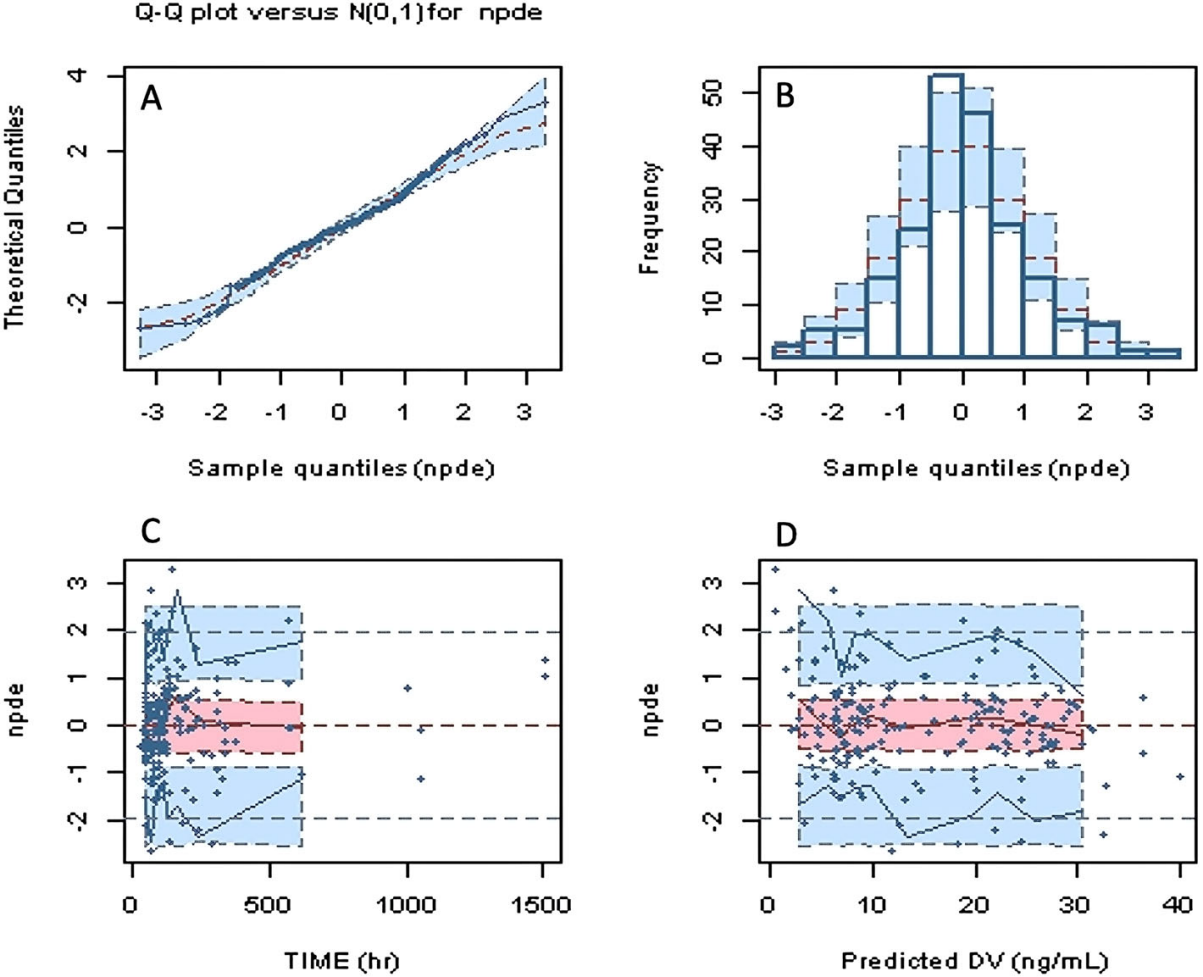
Normalized prediction distribution error (NPDE) for the final model. **a** Quantile-quantile plots of NPDE vs. the expected standard normal distribution; **b** Histogram of NPDE values with the standard normal distribution overlay; **c** Scatter plot of the time vs. NPDE; **d**. Scatterplot of predictions vs. NPDE

In order to calculate the predictive error of each sampling point, we made MPE and MAE diagram for the basic model and the final model. It can be seen from Table 5 that the inter-individual variation of the pharmacokinetic parameters in the final vancomycin PopPK model is significantly reduced compared with the basic model. The NPDE was used to validate the prediction performance of the model. The NPDE was homogeneity of variance and normal distribution according to the quantile plots (Fig. 2), NPDE distribution, and statistical test results. The *t*-test result was 0.566, and the Fisher’s variance test result was 0.859. Such results indicated that the NPDE-preferred model can be used to generate analog data.

At the same time, 1000 data were created for bootstrap validation based on a non-parametric method to obtain 1000 set of model parameters for statistical calculations. If the parameter value was not significantly different, then the model was stable. In this study, more than two model validation methods showed that the final internal model had good stability with superior predictive performance, which can be used to develop individualized dosing regimens for clinical reference.

The performance of the final covariate model was evaluated by visual inspection of diagnostic scatter plots. The robustness of the model was assessed using a nonparametric bootstrap, with replacement, of 1000 NONMEM runs of the final model; the bootstrap median parameter values and the percentile bootstrap 95% intervals were compared with the respective values estimated from the final model. The final model’s 25 estimates were terminated. In addition, the 2.5 to 97.5% confidence interval of CL, V and the estimated results of the covariable parameters were not covered to 0, which met the requirements. All of results are summarized in Table 4.

### Monte Carlo simulation

**Fig. 3:**
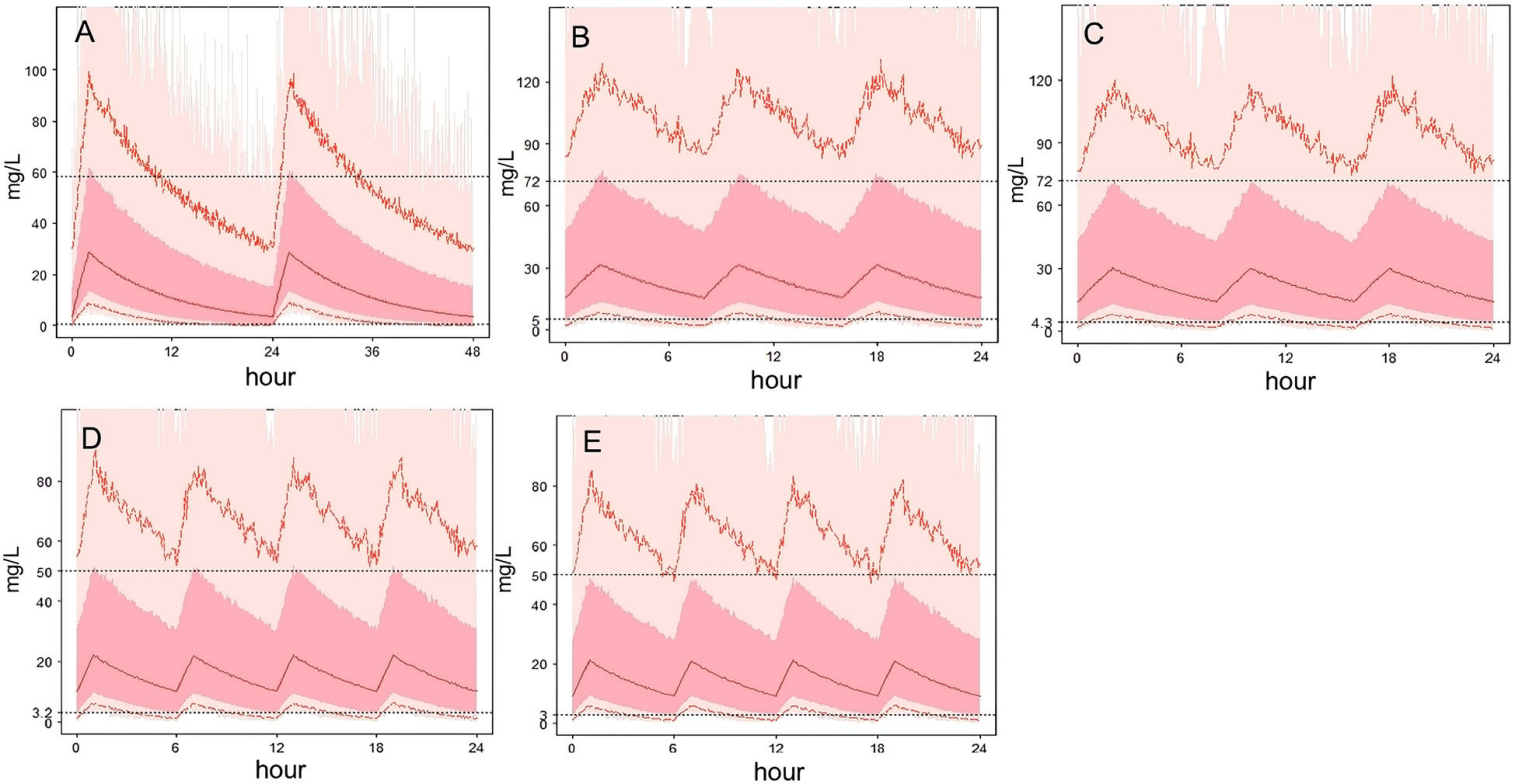
Distribution of the concentration range of vancomycin. Vancomycin concentration–time profile from the Monte Carlo simulation with five typical cases. Deep and light pink colors represent the 5th and 95th percentiles of the simulated data, respectively. Red solid line represents the lower and upper boundaries of the therapeutic range of vancomycin. Horizontal axis: Time after achieving steady state (h); vertical axis: vancomycin concentration (mg/L). **a**. Neonates with 0.95 kg body weight (gestational age: 29 weeks, SCR: 100umol/L, 19 mg every 24 h); **b**. Neonates with 2.4 kg body weight (gestational age: 39 weeks, SCR: 70 μmol/L, 36 mg every 8 h: 0:00, 8:00, and 16:00); **c**. Neonates with 4 kg body weight (gestational age: 39 weeks, 28 days, SCR: 60 μmol/L, 60 mg every 8 h: 0:00,8:00, and 16:00); **d**. Infants with 5 kg body weight (gestational age: 39 weeks, 3 months, SCR: 32 μmol/L, 50 mg every 6 h: 0:00,6:00,12:00, and 18:00); **e**. Infants with 8 kg body weight (gestational age: 39 weeks, 9 months, SCR: 28 μmol/L, 80 mg every 6 h: 0:00, 6:00, 12:00, and 18:00)

The steady state of infants administered with vancomycin was designed, and an analysis was conducted using Monte Carlo simulation to generate simulated data. In children with infection who were taking vancomycin at a steady state, the Monte Carlo simulation generated analog data analysis results showing that trough concentrations of the dosing regimen (15 mg/kg) every 24 h in infants who are 29 weeks premature would be < 5 mg/L, and this did not even meet the dose requirement of 10–15 mg/L [4]. The dosing interval should be shortened; although trough concentrations in 28-day-old neonates were only 4.3 mg/L, the peak concentrations were up to 72 mg/L. Trough concentrations are more likely to be < 5 mg/L when a dose of 10 mg/kg is administered every 6 h in 3–9-month-old infants; thus, the dose should be increased. The clinical application mode of vancomycin has been established and used in individual drug administration in clinical practice, as shown in Fig. 3.

## Discussion

The PK of vancomycin in compartmental model studies, including one-, two-, and three-compartmental models, is controversial. In recent years, one-compartmental model has been successfully used in the study of neonates [17], children [18], adults [19], and different disease statuses of patients [20], which can better meet the needs of the fitting model and estimation of the PK parameters. Because of limited information on drugs, the results preferred two-compartment model as better model to describe the present population. In this study, the PopPK model was successfully established using the one-compartment model as the basis of the structural model for PopPK analysis.

The PopPK model can fully consider the intra- and inter-individual variability; thus, it is advantageous for individual drug delivery. The mixture model was fitted with the simultaneous presence of proportional and additive errors. Factors that influence the PK of vancomycin were selected and investigated, and the effects of weight, SCR, and combined use of drugs were investigated. When screening weight, two methods were used, and meropenem and ceftriaxone as concomitant drugs were also assessed.

Most of the vancomycin in the body were eliminated by the prototype, and the effect of renal function [CLCR or SCR concentrations] on vancomycin clearance rate was investigated. The CLCR data were estimated using the Schwartz formula, and the final model indicated that SCR was an important factor affecting the vancomycin clearance rate. Moreover, the results were consistent with the those reported by Reveilla [21] and Llopis-Salvi [22]. This study first used LZL12B1 as the basic model, adding SCR concentration and CLCR as factors influencing CL, and compared the two covariates to determine which among the two can better describe the CL. Results showed that the addition of SCR concentration and CLCF after the model OFV decreased, and there was a greater reduction in multiplication and addition after the addition of OFV.

Age, weight, and height had an extremely high linear correlation, and height and TP and TP and ALB also had correlation coefficient of > 0.5; thus, attention should be paid to the association between these factors, and such factors must not be added at the same time. In addition, after adding weight, the OFV value decreased by > 100; thus, weight should be retained because it has an important influence on the clearance rate of covariates. Stability and accuracy were in accordance with the requirements.

This study examined the effect on vancomycin elimination rate while administered in combination of other antimicrobial agents (ceftriaxone, meropenem, and gentamicin), furosemide, ibuprofen, and dexamethasone. Based on the basic model, factors of drug combination were investigated, and those that accounted for > 10% of cases were internalized. The effect of drug combination on the clearance rate was investigated using LZL12WS3, respectively in additional method with meropenem and ceftriaxone covariates. Finally, after drawing the Xpose program [15], the distribution of covariates showed a straight line, indicating that the difference between values of covariates and the final model was not statistically different. Thus, the final model only included ceftriaxone.

Because the renal pathway is primarily involved in vancomycin elimination, it is not surprising that the creatinine level is a significant factor of vancomycin clearance. More that 80% of vancomycin intravenously administered are excreted via the urine, and the total body clearance rate is related to renal function. Thus, ceftriaxone may competitively inhibit vancomycin. This study showed that the use of ceftriaxone increases vancomycin clearance rate. Therefore, the protein binding rate of ceftriaxone may be high and have competitive inhibition to vancomycin, and the uncombined vancomycin levels increasingly cleared up [23]. Besides, ceftriaxone may affect the glomerular filtration of vancomycin because the organs of infants were not mature. Similar reports have not yet been published, and the detailed mechanism must be explored in future studies.

Based on the recent guidelines, vancomycin trough concentration of > 10 mg/L was recommended to prevent the development of resistance [24]. Vancomycin trough concentrations of 15–20 mg/L were used to treat serious infections such as endocarditis, osteomyelitis, meningitis, and hospital acquired pneumonia. Predictive performance in the validation step was evaluated by applying the final model to the validation group. The results of this study can be directly applied in clinical practice, and the model can be used to obtain specific PK parameters of a patient to establish vancomycin dosage regimen in patients similar to those in the present study.

The PK parameters of vancomycin in infants with septicemia younger than one year in China were established, and factors influencing the variability of these PK parameters were identified. Inter- and intra-individual variabilities of the PK parameters were determined. The established PK model in this study was stable and had good prediction ability, which can be used to obtain specific PK parameters to establish vancomycin dosage regimen in patients similar to those in the present study and to develop individualized clinical dosing.

## Conclusions

The commended vancomycin dose for infants according to the latest guidelines and instructions is more likely not to obtain target trough concentrations [4, 25]. The median concentration during 29 weeks of gestation was only 3.66 mg/L. We found the dose should be increased and the dosing interval should be decreased by applying final model. As a result, the clinical application of the PopPK model of vancomycin established in the present study can promote the rational use of drugs in clinical practice.

## Data Availability

All data generated or analysed during this study are included in this published article.

## Abbreviations

PopPK: Population pharmacokinetics
MRSA: Methicillin-resistant *Staphylococcus aureus*
PK: Pharmacokinetic
PD: Pharmacodynamic
SCR: Serum creatinine
OFV: Objective function value
NPDE: Normalized prediction distribution errors
TDM: Therapeutic drug monitoring
AST: Aspartate aminotransferase
ALT: Alanine aminotransferase
BUN: Blood Urea Nitrogen
TP: Total protein
